# Optimizing omentoplasty for management of chronic pelvic sepsis by intra‐operative fluorescence angiography: a comparative cohort study

**DOI:** 10.1111/codi.15276

**Published:** 2020-08-17

**Authors:** M. D. Slooter, R. D. Blok, M. A. de Krom, C. J. Buskens, W. A. Bemelman, P. J. Tanis, R. Hompes

**Affiliations:** ^1^ Department of Surgery Cancer Centre Amsterdam Amsterdam UMC University of Amsterdam Amsterdam the Netherlands; ^2^ LEXOR Centre for Experimental and Molecular Medicine Oncode Institute Cancer Centre Amsterdam Amsterdam UMC University of Amsterdam Amsterdam the Netherlands

**Keywords:** fluorescence angiography, indocyanine green, omentoplasty, chronic pelvic sepsis, surgical site infection

## Abstract

**Aim:**

Pelviperineal wound complications frequently occur after salvage surgery for chronic pelvic sepsis despite using an omentoplasty. Sufficient perfusion of the omentoplasty following mobilization is essential for proper healing. This study investigated the impact on short‐term clinical outcomes of fluorescence angiography (FA) using indocyanine green for assessment of omental perfusion in patients undergoing salvage surgery.

**Method:**

This was a comparative cohort study including consecutive patients who underwent combined abdominal and transanal minimally invasive salvage surgery with omentoplasty at a national referral centre for chronic pelvic sepsis between December 2014 and August 2019. The historical and interventional cohorts were defined based on the date of introduction of FA in April 2018. The primary outcome was pelviperineal non‐healing, defined by the presence of any degree of pelviperineal infection at the final postoperative evaluation.

**Results:**

Eighty‐eight patients underwent salvage surgery with omentoplasty for chronic pelvic sepsis, of whom 52 did not have FA and 36 did have FA. The underlying primary disease was Crohn's disease (*n* = 50) or rectal cancer (*n* = 38), with even distribution among the cohorts (*P* = 0.811). FA led to a change in management in 28/36 (78%) patients. After a median of 89 days, pelviperineal non‐healing was observed in 22/52 (42%) patients in the cohort without FA and in 8/36 (22%) patients in the cohort with FA (*P* = 0.051). Omental necrosis was found during reoperation in 3/52 and 0/36 patients, respectively (*P* = 0.266).

**Conclusion:**

After introduction of FA to assess perfusion of the omentoplasty, halving of the pelviperineal non‐healing rate was observed in patients undergoing salvage surgery for chronic pelvic sepsis.

**What does this paper add to the literature?**

The study aim was to evaluate short‐term clinical outcomes in patients undergoing salvage surgery for chronic pelvic sepsis with and without the use of fluorescence angiography (FA) of the omentoplasty. In the cohort with FA, a clinically relevant halving of the pelviperineal non‐healing rate was observed.

## Introduction

Therapy‐refractory pelvic Crohn’s disease and complications after rectal cancer surgery can lead to chronic pelvic sepsis. Salvage surgery without restoration of continuity can offer source control, but persistent pelvic sepsis and pelviperineal wound healing problems might occur, with reported rates up to 60% [1, 2, 3, 4]. Filling of the pelvic cavity by omentoplasty has been proposed to reduce these postoperative infections [3, 4, 5, 6]. Sufficient length of the omentoplasty is one of the requirements for adequate filling of the lower pelvis [7]. Lengthening manoeuvres during creation of the omentoplasty are associated with potential devascularization. If unrecognized intra‐operatively, ischaemic parts of the omentoplasty may result in postoperative necrosis. Omental necrosis, depending on the extent, might require reoperation or is likely to cause secondary infection within the pelvic cavity after longstanding pre‐existing pelvic sepsis, sometimes in combination with prior irradiation.

In a pilot study, the use of intra‐operative fluorescence angiography (FA) using indocyanine green was found to potentially contribute to the diagnosis of omental ischaemia in patients who were treated by salvage surgery and a pedicled omentoplasty [8, 9]. However, the effect on clinical outcomes of the prophylactic resection of poorly perfused omental areas as interpreted by FA is still unknown.

Therefore, the aim of this study was to examine the short‐term clinical outcomes after salvage surgery with omentoplasty for chronic pelvic sepsis with the use of FA, and to compare the results to a historical control cohort of patients who underwent a similar procedure without the use of FA. We hypothesized that a well‐vascularized omentoplasty would lower pelviperineal non‐healing after salvage surgery for pelvic sepsis.

## Method

This study was performed at a national referral centre for chronic pelvic sepsis (Amsterdam UMC, location AMC). All consecutive patients who underwent salvage surgery with omentoplasty for chronic pelvic sepsis by a combined abdominal and transanal minimally invasive (TAMIS) approach between December 2014 and August 2019 were included. FA was introduced as standard clinical practice for assessment of omental perfusion in April 2018. Eligible patients were divided into a historical control cohort before the introduction of FA and an interventional cohort in which FA was routinely used.

Chronic pelvic sepsis originated either from therapy‐refractory pelvic Crohn’s disease or complications after rectal cancer surgery. Therapy‐resistant pelvic Crohn’s disease (referred to as pelvic Crohn’s disease) included poorly controlled proctitis or pouchitis, with or without fistula tracts or anal stenosis. Complications after rectal cancer surgery (referred to as rectal cancer surgery) included chronic sepsis or a chronic presacral sinus. Prior rectal cancer surgery included transanal endoscopic microsurgery, low anterior resection with primary anastomosis, or a Hartmann’s procedure. Patients who underwent salvage surgery and omentoplasty for a different indication other than chronic pelvic sepsis, a non‐TAMIS procedure, and patients who objected to participation were excluded from the study.

Patient data were retrospectively collected using the electronic patient files. The study was approved by the Institutional Review Board of the Amsterdam UMC, location AMC, and the need for written informed consent was waived due to the retrospective nature of the study with no burden for the patient. All patients were sent information concerning the study including an opt‐out letter. When there was no reply within 4 weeks, approval for the use of the data was assumed.

### Surgical procedures

All TAMIS salvage procedures were performed by three colorectal surgeons with extensive experience in minimally invasive surgery and TAMIS procedures. The procedures involved an intersphincteric resection of the rectum following the total mesorectal excision principle if the native rectum was still *in situ* or resection of the colonic conduit/pouch with debridement of the sinus and/or fistula tracts. When not already present, a permanent stoma, either ileostomy or colostomy, was created. The omentoplasty was constructed depending on local anatomy, bulk, length of the omentum and the surgeon's preference. The omentum was mostly pedicled on indication, using either the right or left gastroepiploic artery. The route to the pelvis could be on the right or left side of the middle colic artery, with or without the need for a mesocolic window. After conventional white light assessment of omental perfusion, the decision was made whether or not there was a need for partial omental resection due to ischaemia. From April 2018 onwards, FA was subsequently performed as previously described [8]. FA interpretation was based on the near infrared image (standard setting) and was a consensus between two colorectal surgeons. If the interpretation of FA was inconsistent with the initial intended strategy and additional resection was deemed necessary, this was considered a change in management. Subsequently, areas of the omentum with poor perfusion based on FA only were resected accordingly, and were weighed. During FA, time to the first fluorescent signal was assessed. After FA, the omentum was placed into the pelvic cavity and secured according to the surgeon’s discretion. The perineal wound was closed primarily. Closure of the wound entailed closure of the ischiorectal fat in two separate layers and skin with interrupted vicryl sutures.

### Study outcomes

The primary outcome was pelviperineal non‐healing, defined by any degree of pelviperineal infection that was still present at the last postoperative evaluation. Other outcomes related to the primary outcome included healing without pelviperineal infection, pelviperineal healing by secondary intention, Clavien–Dindo (CD) classification for each postoperative infection, and postoperative omental necrosis.

Healing without infection was defined as an uncomplicated postoperative course considering pelviperineal wound healing. Pelviperineal healing by secondary intention was scored when the course of healing was disrupted by any pelviperineal infection that had resolved before the last evaluation. Pelviperineal infections were defined as any infection in the area of the pelvis or perineum based on available clinical and/or radiological data, and concerned either superficial or deep infections. Superficial infection was superficial cellulitis without pus discharge or abscess objectified by a radiology study. Deep infection was scored when a perineal or presacral abscess occurred with or without unhealed fistula tracts. The definition of omental necrosis was based on confirmed necrosis of the omentoplasty during reoperation.

Secondary outcomes included postoperative hospital stay, hospital readmissions, total hospital stay during the study period, 90‐day postoperative mortality, operative details including the technique of the omentoplasty, and FA details. FA details entailed change in management related to the omentoplasty due to use of the technique, additional resected omental tissue (in grams) and the time between indocyanine green injection and fluorescent enhancement in the omentoplasty and the visible demarcation line (in seconds).

### Statistics

Categorical data were presented as number of cases and percentages. Continuous data, when normally distributed, were shown as mean and standard deviation (±SD) or, when not normally distributed, as median and interquartile range (IQR). Comparison of categorical variables was done using the chi‐squared or Fisher’s exact test. Comparison of continuous variables was done with a *t* test or Mann–Whitney *U* test, according to distribution. Univariate binary logistic regression analyses were performed to ascertain whether the technique of omentoplasty had an effect on the occurrence of deep pelviperineal infection. All analyses were performed using IBM (IBM Corporation, Armonk, New York, USA) spss Statistics (version 25.0.0).

## Results

**Figure 1:**
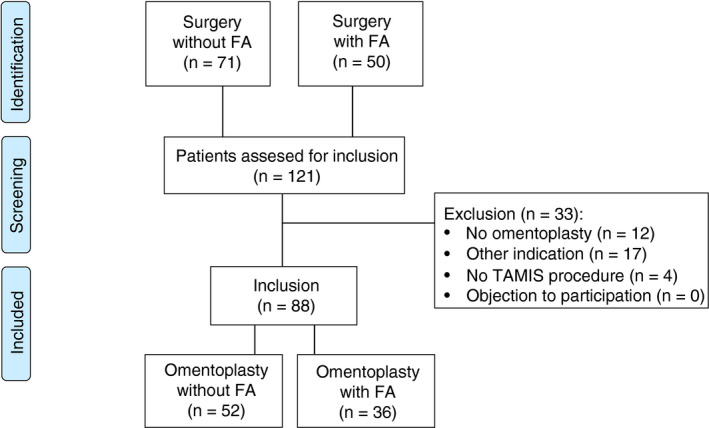
Flow diagram inclusion process.

Between December 2014 and August 2019, a total of 88 patients underwent salvage surgery with omentoplasty for chronic pelvic sepsis. The historical control cohort without FA comprised 52 patients, and 36 patients were included in the interventional cohort in which the omentoplasty was routinely assessed using FA. The inclusion process is summarized in Fig. 1.

Baseline characteristics for the two cohorts are presented in Table 1. Overall, the median age was 55 years (IQR 40–69). Fifty patients had a history of therapy‐refractory pelvic Crohn’s disease (57%), and 38 patients had undergone complicated surgery for rectal cancer. There was no statistical difference between the cohorts in age, sex, body mass index, American Society of Anesthesiologists score, and underlying primary disease. The cohort with FA included fewer patients with a history of vascular disease (3% *vs* 19%, *P* = 0.024). A summary of prior therapies and procedures is presented in Table S1. Almost all patients in the rectal cancer surgery group underwent neoadjuvant radiotherapy before the initial surgery (36/38).

**Table 1:** Baseline characteristics.

	Omentoplasty without FA (*n* = 52)	Omentoplasty with FA (*n* = 36)	*P*
Age, years, median (IQR)	55 (39–69)	56 (40–69)	0.763
Male sex	29/52 (56)	16/36 (44)	0.296
BMI, kg/m^2^, mean ± SD	25.2 ± 3.9	26.7 ± 5.8	0.133
ASA classification ≥ 3	13/52 (25)	10/36 (28)	0.771
Active smoker	13/52 (25)	7/36 (19)	0.541
Comorbidity			
Diabetes mellitus*	5/52 (10)	3/36 (8)	1.000
Vascular disease^†^	10/52 (19)	1/36 (3)	*0.024*
Pulmonary disease^‡^	3/52 (6)	2/36 (6)	1.000
Pelvic sepsis originating from			
Pelvic Crohn's disease	29/52 (56)	21/36 (58)	0.811
Rectal cancer surgery	23/52 (44)	15/36 (42)	0.811

Data shown in *n* (%) unless otherwise stated. Italic values indicate when *P* < 0.05.

ASA, American Society of Anesthesiologists; BMI, body mass index; FA, fluorescence angiography; IQR, interquartile range.

* Diabetes mellitus type 1, 2 and steroid induced.

^†^ Including brain infarction, myocardial infarction, peripheral vascular disease.

^‡^ Including chronic obstructive pulmonary disease and asthma.

### Surgical procedures

The operative procedures and details are presented in Table 2. A full laparoscopic approach for the abdominal phase was more often used in the cohort with FA (69%) than in cohort without FA (39%; *P* = 0.004). The omentoplasty was more frequently pedicled in the cohort with FA (*P* = 0.012), and, when pedicled, it was more often pedicled on the right gastroepiploic artery (*P* = 0.008). The various routes of the omentoplasty to the lower pelvis are described in Table 2. Partial ischaemia of the omentoplasty was noticed intra‐operatively by conventional white light in nine (17%) out of 52 patients in the cohort without FA and in four (12%) out of 36 patients in the cohort with FA (*P* = 0.421). Specific characteristics of FA are demonstrated in Table 3. In the cohort with FA, change in management due to FA occurred in 28 (78%) out of 36 cases and led to an additional resection of a median of 37 g (IQR 17–100) of omentum. In one case, additional resection resulted in insufficient bulk of omentum for pelvic filling, and therefore a gluteal turnover flap was created for additional filling and perineal closure [10, 11]. Use of FA and subsequent management took a median of 7 min (IQR 3–13). There were no significant differences in the occurrence of intra‐operative complications.

**Table 2:** Surgical procedure.

	Omentoplasty without FA (*n* = 52)	Omentoplasty with FA (*n* = 36)	*P*
Stoma present at start of surgery	47/52 (90)	33/36 (92)	1.000
Surgical procedure – pelvic Crohn's disease			
IS TME proctectomy*	21/29 (72)	17/21 (81)	0.485
IS pouch excision	3/29 (10)	2/21 (10)	1.000
IS resection anastomosis	1/29 (3)	2/21 (10)	0.565
Excision mesorectum^†^	4/29 (14)	0/21 (0)	0.129
Surgical procedure – rectal cancer surgery			
IS TME proctectomy*	11/23 (48)	4/15 (27)	0.192
IS resection anastomosis	12/23 (52)	11/15 (73)	0.192
Abdominal approach			
Laparoscopy	20/52 (39)	25/36 (69)	*0.004*
Laparoscopy with conversion to open	6/52 (12)	4/36 (11)	1.000
Hand‐assisted laparoscopy	3/52 (6)	1/36 (3)	0.642
Open^‡^	23/52 (44)	6/36 (17)	*0.007*
Technique of omentoplasty			
Non‐pedicled	20/52 (39)	5/36 (14)	*0.012*
Left gastroepiploic pedicle	24/32 (75)	13/31 (42)	*0.008*
Right gastroepiploic pedicle	8/32 (25)	18/31 (58)	*0.008*
Omentoplasty route to pelvis			
Left paracolic gutter	11/52 (21)	7/36 (19)	0.845
Along Treitz, after subtotal colectomy	19/52 (37)	10/36 (28)	0.390
Left to middle colic artery, through mesocolic window	17/52 (33)	5/36 (14)	*0.045*
Right to middle colic artery, no mesocolic window	3/52 (6)	2/36 (6)	1.000
Right to middle colic artery, through mesocolic window	2/52 (4)	12/36 (33)	*< 0.001*
Partial ischaemia of the omentoplasty	9/52 (17)	32/36 (89)	*< 0.001*
Detected by white light assessment	9/52 (17)	4/36 (12)	0.421
Primary perineal wound closure	52/52 (100)	35/36 (97)	0.409
Intra‐operative complication(s)^§^	6/52 (12)	6/36 (17)	0.539
Total operative time, min, median (IQR)	240 (201–310)	258 (231–328)	*0.047*
Surgical drain in pelvis	44/52 (85)	33/36 (92)	0.514

Data shown in *n* (%) unless otherwise stated. Italic values indicate when *P* < 0.05.

FA, fluorescence angiography; IQR, interquartile range; IS, intersphincteric; TME, total mesorectal excision.

* Including proctocolectomy, proctectomy and rest‐proctectomy.

^†^ Completion total mesorectal excision for Crohn's disease.

^‡^ Midline laparotomy or Pfannenstiel.

^§^ Including difficulties with intubation, full thickness perforation, extensive adhesiolyse, ureter injury, bleeding or gallbladder defect.

**Table 3:** Fluorescence angiography.

	Omentoplasty with FA (*n* = 36)
Dose ICG, mg	8.0 (7.5–10)
Time ICG injection to first enhancement omentoplasty*, s	19 (12–32)
Time ICG injection to visible demarcation line*, s	37 (28–45)
Change in management, *n* (%)	28/36 (78)
Resected omental fat, g	37 (17–100)
Additional surgical time due to FA^†^, min	7 (3–13)

Data shown in median (interquartile range) unless otherwise stated.

ICG, indocyanine green; FA, fluorescence angiography.

* Data known for 28/36 patients.

^†^ Data known for 29/36 patients.

### Postoperative course

Outcomes were evaluated with a median follow‐up of 89 days (IQR 49–101), with no significant difference in follow‐up between the cohorts.

Postoperative outcomes are demonstrated in Table 4. In the cohort with FA, less pelviperineal non‐healing was observed at the last postoperative evaluation [8/36 (22%) *vs* 22/52 (42%), *P* = 0.051]. All the 30 patients with pelviperineal non‐healing had persisting deep infections, and 16 (53%) patients were treated for pelvic Crohn’s disease.

**Table 4:** Postoperative outcomes.

	Omentoplasty without FA (*n* = 52)	Omentoplasty with FA (*n* = 36)	*P*
Healed at last postoperative evaluation	30/52 (58)	28/36 (78)	0.051
Healing without pelviperineal infection	27/52 (52)	24/36 (67)	0.168
Pelviperineal infection healed at secondary intent	3/52 (6)	4/36 (11)	0.438
Observed pelviperineal infection*	25/52 (48)	12/36 (33)	0.168
Superficial infection	0/52 (0)	1/36 (3)	0.409
Deep infection	25/52 (48)	11/36 (30)	0.100
In combination with unhealed fistula tracts	9/52 (17)	2/36 (6)	0.188
CD ≥ 2	17/25 (68)	9/12 (75)	1.000
CD 2	9/25 (36)	2/12 (17)	0.279
CD 3a	3/25 (12)	3/12 (25)	0.367
CD 3b	3/25 (12)	4/12 (33)	0.183
CD 4	2/25 (8)	0/12 (0)	1.000
Hospital stay			
Postoperative hospital stay, days, median (IQR)	7 (4–11)	6 (4–10)	0.588
Hospital readmission	6/52 (12)	9/36 (25)	0.099
Total in hospital stay, days, median (IQR)	7 (5–12)	7 (4–12)	0.959

Data shown in *n* (%) unless otherwise stated.

CD, Clavien–Dindo classification; FA, fluorescence angiography; IQR, interquartile range.

* Within study period.

Overall, pelviperineal infections at any time during the postoperative course occurred in 25 (48%) out of 52 patients in the cohort without FA and in 12 (33%) out of 36 patients in the cohort with FA (*P* = 0.168). Of these, one superficial and six deep pelviperineal infections healed by secondary intention.

Omental necrosis confirmed during reoperation occurred in three out of 52 patients in the cohort without FA, and none of the patients in the cohort with FA had omental necrosis (*P* = 0.266). All cases of omental necrosis occurred in the group after rectal cancer surgery. The proportion of complications with a CD score above 2 was eight (32%) out of 25 in the cohort without FA compared to seven (58%) out of 12 in the cohort with FA (*P* = 0.164). All reoperations in the cohort without FA were performed for omental necrosis. In the cohort with FA, reoperations were performed for persistent pelvic sepsis but with a viable omentoplasty (confirmed during reoperation by both conventional white light and FA assessment).

Results separated for pelvic Crohn’s disease and rectal cancer surgery patients and for the technique of omentoplasty (non‐ *vs* pedicled; left *vs* right pedicled) are demonstrated in Tables S2A and S2B, respectively. The effect on pelviperineal non‐healing was comparable to the overall group (Table 4) for pelvic Crohn’s disease and rectal cancer surgery subpopulations, and for pedicled and non‐pedicled omentoplasty. A decrease in overall pelviperineal infections was observed in the FA cohort for non‐ and left pedicled omentoplasties compared to the overall group, but this effect was not observed for right pedicled omentoplasties. A *post hoc* univariate regression analysis on the effect of a left *vs* right pedicled omentoplasty on deep pelviperineal infection showed an OR of 9.800 (95% CI 1.036–92.696, *P* = 0.046) when no FA was used. After the use of FA, this risk had an OR of 2.187 (95% CI 0.452–10.576, *P* = 0.330).

There was no significant statistical difference in postoperative hospital stay, readmission rate and total hospital stay during the study period. The mortality rate within 90 days postoperatively was two (4%) out of 52 patients in the cohort without FA and 0 out of 36 patients in the cohort with FA.

## Discussion and conclusions

This study evaluated the potential clinical value of FA of the omentoplasty for pelvic filling in patients with chronic pelvic sepsis who underwent salvage surgery. Patients in the cohort with FA demonstrated a trend towards less deep pelviperineal infections, and importantly no omental necrosis. This resulted in less pelviperineal non‐healing at the last postoperative evaluation with a non‐ statistical significance, but potential clinical relevance. These results support to some extent the hypothesis that FA and subsequent resection of poorly perfused omentum may prevent postoperative omental necrosis with risk of secondary infection.

Postoperative omental necrosis has been reported in the literature in merely 2%–4% of cases [12, 13, 14], but there is probably an important underreporting of partial omental necrosis in the case of secondary infection that is not being attributed to the omentoplasty and without the need for reoperation. The idea that secondary infection can occur as a complication of occult omental necrosis might be supported by the observed lower rates of deep infections after the use of FA.

However, there is a clear discrepancy between the change in management rate in the cohort with FA and reduction of postoperative omental necrosis, even when adding the reduction of deep infections that may have been secondary to occult omental necrosis. In the current study, omental perfusion was judged to be partially compromised in most patients (89%) after use of both conventional white light and FA assessment. The percentage of intra‐operative detection of partial omental ischaemia by conventional assessment (17% and 12% in the two cohorts, respectively) corresponds to reported rates in the literature [14]. In the cohort with FA, resection of ischaemic parts of the omentoplasty was performed according to FA findings in an additional 78%, which is in line with the observation from a previous pilot study [8].

Several explanations can be considered for the observed discrepancy. First, the interpretation of FA remains subjective. There are no thresholds to guide whether the perfusion as assessed by FA is sufficient or not. This means that what has subjectively been interpreted as ‘poor perfusion’ may actually be sufficient and not lead to omental necrosis. Another explanation may be related to the fact that probably not all cases of omental necrosis will give rise to secondary infection or healing problems. Partial necrosis of fatty tissue has been described to be associated with non‐specific complaints such as low grade fever or abdominal pain, and is often self‐limiting [15]. In a few cases in the cohort with FA, only small parts of poorly perfused omentum (< 10 g) were recorded as a change in management. Probably the effect of prophylactic resection of such small parts is limited and may not be clinically relevant. The observed discrepancy between deep infections and change in management might indicate that the use of FA leads to overtreatment. However, this overtreatment might be acceptable considering minimal additional intervention and operating time, especially in this complex patient group of chronic pelvic sepsis in which it is mandatory to fill the pelvic cavity with well‐vascularized omental tissue. Furthermore, this approach led to only one case of insufficient omental bulk for pelvic filling in this study, where additional reconstruction using a perineal flap was necessary.

An interesting finding of this study was that FA had less impact when a right pedicled omentoplasty was performed. When comparing the results between left and right pedicled omentoplasties, the differences in outcomes were larger in patients in the cohort without FA. This might be explained by the fact that the possible hampered perfusion after a left pedicled omentoplasty in patients who received FA was corrected by the partial omentectomy due to the FA assessment. The hypothesis that the right gastroepiploic artery is the stronger one as the diameter is bigger is not novel [16], but this study provides new data to suggest that a pedicle on the right artery might be associated with lower risk of secondary infection.

There are some limitations to this study. First, it was implicated that non‐healing was mostly due to (partial) omental necrosis. Although omentum necrosis is expected to be a major cause of non‐healing problems, other major causes include inadequate debridement, infected haematoma, any necrotic tissue, undrained fluid, and patient risk factors including duration and extent of pre‐existing infection and diabetes mellitus. In the historic cohort a higher rate of cardiovascular comorbidity was observed. Although this patient factor could have led to bias in the results, cardiovascular comorbidity is expected to play a minor role according to our own experience. Secondly, the sample size was still relatively small and the study may have been subject to confounding factors due to the retrospective design with a historical comparison. The results could be confounded by change in surgical technique of omentoplasty and change in approach regarding management of complications over time. Comparing the interventional with the historical cohort, a laparoscopic approach was used more often and more pedicled omentoplasties were performed. Patients in the cohort with FA could potentially have received better quality omentoplasties with sufficient length and bulk in the pelvic cavity. However, the added value of FA could be larger for pedicled omentoplasties, particularly left omentoplasties. In this study, the effect of FA was also noticed for non‐pedicled omentoplasties. An explanation might be the change in anatomy after prior procedures leading to ligation of omental arterial branches. In the FA cohort, less pelviperineal infections and no omental necrosis were observed; however, more reoperations were performed. This could be explained by more active surgical management of persistent sepsis over time. Reoperations could also have contributed to the primary outcome of less pelviperineal non‐healing in both cohorts. Furthermore, follow‐up included only short‐term complications, and a proportion of patients were lost to follow‐up before 90 days postoperatively. Reasons for loss to follow‐up usually concerned patient’s return to the referring hospital when there was an uncomplicated postoperative course, and usually the referring hospital reported when new complications occurred. Also, pelviperineal infections are difficult to distinguish retrospectively into pelvic and perineal infections, which is the reason for combining the two in one denominator. Finally, we included a heterogeneous patient population with chronic pelvic sepsis originating from both therapy‐refractory pelvic Crohn’s disease and after rectal cancer surgery. However, the effect of FA was similar for both subgroups.

Nevertheless, this comparative study adds to the literature as there is no available data yet on the effect of FA on an omentoplasty in patients undergoing salvage surgery for chronic pelvic sepsis, and the results strongly support future research. Future studies should be carried out prospectively in a larger cohort to confirm our results. Also research on FA of omentoplasty should focus on the determination of fluorescent thresholds to point out adequate perfused tissue.

## Funding

RH: unrestricted grant and materials Stryker European Operations B.V.

## Ethics approval

The protocol of the study was approved by the Institutional Review Board of the Amsterdam UMC, location AMC (W19_065), and confirmed that the Medical Research lnvolving Human Subjects Act (WMO) did not apply.

## Patients consent

The need for written informed consent was waived due to the retrospective nature of the study with no burden for the patient. All patients were sent information concerning the study including an opt‐out letter. When not replied to within 4 weeks, approval for the use of the data was assumed.

## Supporting information


**Table S1.** Prior therapy.
**Table S2.** Subgroup analysis.

## Data Availability

Data available on request from the authors.
