# Supplementary material for: Optimizing omentoplasty for management of chronic pelvic sepsis by intra‐operative fluorescence angiography: a comparative cohort study
Source: Colorectal Dis. 2020 Aug 17;22(12):2252–9. doi: 10.1111/codi.15276 (PMC7818129; doi:10.1111/codi.15276)
Supplement: Supplementary file 1 — Table S1. Prior therapy. Table S2. Subgroup analysis. [file CODI-22-2252-s001.docx]

**Supplementary Tables**

Supplementary Table 1. Prior therapy

Supplementary Table 2. Subgroup analysis

A. Comparing results for Therapy-refractory pelvic Crohn's Disease vs Complications after rectal cancer surgery

B. Comparing results for technique of omentoplasty

**Supplementary table 1**: Prior therapy

|  | **Omentoplasty without FA**  **(n = 52)** | **Omentoplasty with FA**  **(n = 36)** | **P** |
| --- | --- | --- | --- |
| **Therapy-refractory pelvic Crohn's Disease** | | | |
| **Therapy** |  |  |  |
| Active steroid use¹ | 7/29 (24) | 3/21 (14) | 0.488 |
| Biologicals² | 15/29 (52) | 12/21 (57) | 0.704 |
| **Prior pelvic surgery** | 21/29 (72) | 15/21 (71) | 0.939 |
| Fistula treatment | 9/29 (31) | 11/21 (52) | 0.128 |
| Abscess drainage | 12/29 (41) | 5/21 (24) | 0.196 |
| IPAA | 3/29 (6) | 2/21 (10) | 1.000 |
| Non-restorative proctectomy | 5/29 (17) | 3/21 (14) | 1.000 |
| LAR | 2/29 (7) | 1/21 (5) | 1.000 |
| **Complications after rectal cancer surgery** | | | |
| **Neo-adjuvant therapy** | 21/23 (91) | 15/15 (100) | 0.509 |
| Short-course radiotherapy | 15/23 (65) | 6/15 (40) | 0.126 |
| Long-course chemoradiotherapy | 5/23 (22) | 8/15 (53) | *0.045* |
| Unknown schedule | 1/23 (4) | 1/15 (7) | 1.000 |
| **Initial rectal cancer surgery** | 23/23 (100) | 15/15 (100) | - |
| TEM | 1/23 (4) | 0/15 (0) | 1.000 |
| LAR | 18/23 (78) | 14/15 (93) | 0.371 |
| Hartmann's procedure | 7/23 (13) | 1/15 (7) | 0.114 |
| **Re-operations after initial rectal surgery** | 10/23 (44) | 7/15 (47) | 0.847 |
| Re-do anastomosis | 3/23 (13) | 2/15 (13) | 1.000 |
| Abscess drainage | 10/23 (44) | 5/15 (33) | 0.537 |

Data shown in n (%) unless otherwise stated.

FA Fluorescence Angiography, IPAA Ileal Pouch-Anal Anastomosis, LAR Low Anterior Resection, TEM Transanal Endoscopic Microsurgery

¹ >20mg/day

² Within 12 weeks prior to surgery

**Supplementary table 2**: Subgroup analyses

**Supplementary table 2A**: Comparing results for Therapy-refractory pelvic Crohn's Disease vs Complications after rectal cancer surgery

|  | **Therapy-refractory pelvic Crohn's Disease** | | | **Complications after rectal cancer surgery** | | |
| --- | --- | --- | --- | --- | --- | --- |
|  | **Omentoplasty without FA**  **(n = 29)** | **Omentoplasty**  **with FA**  **(n = 21)** | **P** | **Omentoplasty without FA**  **(n = 23)** | **Omentoplasty with FA**  **(n = 15)** | **P** |
| **Healed at end of follow-up** | 17/29 (59) | 17/21 (81) | 0.095 | 13/23 (57) | 11/15 (73) | 0.294 |
| Healing without pelviperineal infection | 16/29 (55) | 14/21 (67) | 0.413 | 11/23 (48) | 10/15 (67) | 0.254 |
| Pelviperineal infection healed at secondary intent | 1/29 (3) | 3/21 (14) | 0.297 | 2/23 (9) | 1/15 (7) | 1.000 |
| **Observed pelviperineal infection*** | 13/29 (45) | 7/21 (33) | 0.413 | 12/23 (52) | 5/15 (33) | 0.254 |
| Deep infection | 13/29 (45) | 6/21 (29) | 0.242 | 12/23 (52) | 5/15 (29) | 0.254 |
| *In combination with unhealed fistula tract* | 5/29 (17) | 1/21 (5) | 0.380 | 4/23 (17) | 1/15 (7) | 0.630 |
| CD ≥2 | 7/13 (54) | 6/7 (86) | 0.329 | 10/12 (83) | 3/5 (60) | 0.538 |

**Supplementary table 2B**: Comparing results for technique of omentoplasty

|  | **Non-pedicled omentoplasty** | | | **Pedicled omentoplasty** | | | **Left pedicled omentoplasty** | | | **Right pedicled omentoplasty** | | |
| --- | --- | --- | --- | --- | --- | --- | --- | --- | --- | --- | --- | --- |
|  | **Omentoplasty without FA**  **(n = 20)** | **Omentoplasty with FA**  **(n = 5)** | **P** | **Omentoplasty without FA**  **(n = 32)** | **Omentoplasty with FA**  **(n = 31)** | **P** | **Omentoplasty without FA**  **(n = 24)** | **Omentoplasty with FA**  **(n = 13)** | **P** | **Omentoplasty without FA**  **(n = 8)** | **Omentoplasty with FA**  **(n = 18)** | **P** |
| **Healed at end of follow-up** | 12/20 (50) | 4/5 (80) | 0.621 | 18/32 (56) | 24/31 (77) | 0.075 | 11/24 (46) | 8/13 (62) | 0.478 | 7/8 (88) | 16/18 (89) | 1.000 |
| Healing without pelviperineal infection | 10/20 (50) | 3/5 (60) | 1.000 | 17/32 (53) | 21/31 (68) | 0.236 | 10/24 (42) | 7/13 (54) | 0.478 | 7/8 (88) | 14/18 (78) | 1.000 |
| Pelviperineal infection healed at  secondary intent | 2/20 (10) | 1/5 (20) | 0.504 | 1/32 (3) | 3/31 (10) | 0.355 | 1/24 (4) | 1/13 (8) | 1.000 | 0/8 (0) | 2/18 (11) | 1.000 |
| **Observed pelviperineal infection*** | 10/10 (50) | 2/5 (40) | 1.000 | 15/32 (47) | 10/31 (32) | 0.236 | 14/24 (58) | 6/13 (46) | 0.478 | 1/8 (13) | 4/18 (22) | 1.000 |
| Deep infection | 10/10 (50) | 2/5 (40) | 1.000 | 15/32 (47) | 9/31 (29) | 0.145 | 14/24 (58) | 5/13 (39) | 0.248 | 1/8 (13) | 4/18 (22) | 1.000 |
| *In combination with unhealed fistula*  *tract* | 4/20 (20) | 0/5 (0) | 0.549 | 5/32 (16) | 2/31 (7) | 0.426 | 4/24 (17) | 2/13 (15) | 1.000 | 1/8 (13) | 0/18 (0) | 0.308 |
| CD ≥2 | 6/20 (30) | 1/5 (20) | 1.000 | 11/32 (34) | 8/31 (26) | 0.459 | 10/14 (72) | 5/6 (83) | 1.000 | 1/1 (100) | 3/4 (75) | 1.000 |
